# Raw Sheep Milk as a Reservoir of Multidrug-Resistant *Staphylococcus aureus*: Evidence from Traditional Farming Systems in Romania

**DOI:** 10.3390/antibiotics14080787

**Published:** 2025-08-02

**Authors:** Răzvan-Dragoș Roșu, Adriana Morar, Alexandra Ban-Cucerzan, Mirela Imre, Sebastian Alexandru Popa, Răzvan-Tudor Pătrînjan, Alexandra Pocinoc, Kálmán Imre

**Affiliations:** Department of Animal Production and Veterinary Public Health, Faculty of Veterinary Medicine, University of Life Sciences “King Mihai I” from Timisoara, 300645 Timisoara, Romania; razvan.rosu@usvt.ro (R.-D.R.); adrianamorar@usvt.ro (A.M.); alexandracucerzan@usvt.ro (A.B.-C.); mirela.imre@usvt.ro (M.I.); sebastian.popa@usvt.ro (S.A.P.); razvan.patrinjan.fmv@usvt.ro (R.-T.P.); alexandra.pocinoc@usvt.ro (A.P.)

**Keywords:** *S. aureus*, sheep milk, antimicrobial resistance, methicillin-resistant, traditional dairy farming, Romania

## Abstract

**Background/Objectives**: *Staphylococcus aureus* is a major pathogen of concern in raw milk due to its potential to cause foodborne illness and its increasing antimicrobial resistance (AMR). In Romania, data on the occurrence and resistance patterns of *S. aureus* in raw sheep milk from traditional farming systems remain limited. This study investigated the presence and antimicrobial resistance of *S. aureus* in 106 raw sheep milk samples collected from traditional farms in the Banat region of western Romania. **Methods**: Coagulase-positive staphylococci (CPS) were enumerated using ISO 6888-1:2021 protocols. Isolates were identified at the species level using the Vitek 2 system and molecularly confirmed via PCR targeting the 16S rDNA and *nuc* genes. Methicillin resistance was assessed by detecting the *mecA* gene. Antimicrobial susceptibility was determined using the Vitek 2 AST-GP79 card. **Results**: CPS were detected in 69 samples, with *S. aureus* confirmed in 34.9%. The *mecA* gene was identified in 13.5% of *S. aureus* isolates, indicating the presence of methicillin-resistant *S. aureus* (MRSA). Resistance to at least two antimicrobials was observed in 97.3% of isolates, and 33 strains (89.2%) met the criteria for multidrug resistance (MDR). The most frequent MDR phenotype involved resistance to lincomycin, macrolides, β-lactams, tetracyclines, and aminoglycosides. **Conclusions**: The high prevalence of *S. aureus*, including MRSA and MDR strains, in raw sheep milk from traditional farms represents a potential public health risk, particularly in regions where unpasteurized dairy consumption persists. These findings underscore the need for enhanced hygiene practices, prudent antimicrobial use, and AMR monitoring in small-scale dairy systems.

## 1. Introduction

Sheep milk plays a significant role in the agri-food economy and traditional diets of Romania, particularly in the Banat region, where extensive sheep farming and artisanal cheese production are long-standing practices [1,2]. In addition to its cultural and economic importance, sheep milk is recognized for its rich nutritional profile, being notably higher in protein, essential amino acids, calcium, and vitamins such as B12 and riboflavin compared to cow or goat milk [3,4,5]. It also contains higher levels of beneficial short- and medium-chain fatty acids, which have been associated with improved digestion and cardiovascular health [3,4]. In many rural and peri-urban areas of the Banat region, raw sheep milk and unpasteurized dairy products (such as traditional cheeses) are commonly consumed, often directly from farms or local markets. Household-level consumption of raw milk can vary from 200 to 500 mL per day per person, particularly during peak lactation months. This cultural preference for raw dairy increases the likelihood of human exposure to milk-borne pathogens and resistant bacteria, particularly in the absence of regulatory controls [2,5]. Despite these advantages, the widespread consumption of raw or minimally processed sheep milk and dairy products, often produced under variable hygienic conditions, raises legitimate concerns regarding microbiological safety and the potential transmission of foodborne pathogens and antimicrobial-resistant bacteria [6,7].

Among the bacteria of concern in raw milk, *S. aureus* is of particular importance due to its dual impact on both animal health and public safety. Taxonomically, *S. aureus* belongs to the genus *Staphylococcus*, within the family *Staphylococcaceae*, order *Bacillales*, class *Bacilli*, and phylum *Firmicutes* [8]. As a Gram-positive, facultatively anaerobic coccus, it typically appears microscopically in grape-like clusters. On solid culture media, it forms smooth, circular, golden-yellow colonies, a characteristic reflected in its Latin name “aureus” (golden). It exhibits β-hemolysis on blood agar and is both catalase- and coagulase-positive, distinguishing features that aid in its identification [8,9].

In dairy animals, *S. aureus* is a leading cause of clinical and subclinical mastitis, contributing to decreased milk yield, altered milk composition, and significant economic losses for farmers [10]. Its pathogenic potential is underpinned by a complex arsenal of virulence factors, including adhesins, immune evasion proteins, cytotoxins, and enzymes, which enable it to persist within the host, evade immune defenses, and establish both localized and systemic infections [8]. This combination of pathogenicity, environmental resilience, and increasing antimicrobial resistance makes *S. aureus* a critical target for both veterinary and food safety interventions [10].

From a public health perspective, its presence in raw milk is concerning due to its ability to produce heat-stable staphylococcal enterotoxins, which can cause foodborne intoxications even after pasteurization [11]. Moreover, the increasing detection of antibiotic-resistant and methicillin-resistant *S. aureus* (MRSA) strains in livestock systems worldwide, including in Romania, exacerbates the challenge of controlling infections and limiting their transmission through the food chain [12,13].

A recent study conducted in western Romania has identified *S. aureus* in over one-third of coagulase-positive staphylococci (CPS) isolated from traditional cheese products, with enterotoxin-producing strains also being detected [14]. In this study, a significant proportion of isolates exhibited resistance to commonly used antimicrobials (e.g., penicillin, amikacin, and enrofloxacin), and nearly half demonstrated multidrug resistance (MDR) phenotypes. Furthermore, the detection of MRSA strains in bovine mastitis milk in the same region highlights the zoonotic potential and clinical relevance of antimicrobial-resistant *S. aureus* in dairy environments [15].

Parallel investigations in the Banat region have also drawn attention to the presence of another important foodborne pathogen, *Escherichia coli*, in raw sheep milk [2]. These findings underscore the microbiological vulnerability of raw sheep milk and suggest the need for comprehensive monitoring of multiple bacterial hazards in this matrix. While *E. coli* contamination is often linked to poor hygienic practices during milking, *S. aureus* contamination can also arise from intramammary infections, making its presence a concern not only for food safety but also for animal welfare and antimicrobial stewardship [16,17].

Despite the well-documented risks associated with *S. aureus* in various dairy species and products, data on its occurrence and antimicrobial resistance profile in raw milk in Romania remain limited. A recent study conducted in the Banat region has provided initial insights into the presence of *S. aureus* in this matrix, highlighting the need for broader surveillance and more comprehensive investigations into its epidemiology and public health significance [16].

Given the growing evidence of microbial and antimicrobial resistance hazards in sheep milk from the Banat region, the present study aimed to investigate the occurrence of *S. aureus* in raw sheep milk and to characterize its antimicrobial resistance patterns. This research seeks to contribute to a more integrated understanding of foodborne risks in traditional dairy systems and to support the development of targeted interventions to protect public health.

## 2. Results

Out of the 106 raw sheep milk samples collected across the Banat region over two consecutive lactation seasons (2024 and 2025), CPS were detected in 69 samples, reflecting an overall prevalence of 65.1% (95% Confidence Interval: 55.6–73.5). Seasonal variation was minimal, with monthly CPS detection rates ranging from 14 (60.9%) positive samples in April 2025 to 19 (70.4%) positive samples in May 2024 (Table 1). Quantitative CPS counts in positive samples ranged from 1.00 to 4.91 log CFU/g, with an overall mean contamination level of 2.20 ± 0.20 log CFU/g.

Species-level identification using the Vitek 2 system revealed the presence of *S. aureus* in 37 of the CPS-positive samples, corresponding to 53.6% of CPS-positive samples and 34.9% of all tested milk samples. The monthly frequency of *S. aureus* detection among CPS-positive samples showed moderate variation, ranging from 8 (42.1%) samples in May 2024 to 14 (58.3%) samples in May 2025, but no statistically significant differences were observed between months (*p* > 0.05).

The presence of the 16S rDNA gene confirmed genus-level identity as *Staphylococcus* spp., while detection of the *nuc* gene—a species-specific marker—provided molecular confirmation of the Vitek2 biochemical identification of *S. aureus.* Genotypic screening for the *mecA* gene, a marker of methicillin resistance, identified five methicillin-resistant *S. aureus* (MRSA) isolates, yielding a prevalence of 13.5% among *S. aureus* strains and 4.7% across all samples. The *mecA* gene was detected sporadically across sampling months, with the highest monthly proportion noted in April 2025 (25.0%), followed by April 2024 (14.3%) and May 2025 (14.3%), while no MRSA strains were found in May 2024.

These results underscore the widespread occurrence of CPS and *S. aureus* in raw sheep milk from traditional farms in the Banat region, with a concerning proportion exhibiting methicillin resistance. The consistent detection of *S. aureus* across seasons and the presence of MRSA strains highlight a public health risk and the need for improved hygiene and AMR monitoring on farms.

Out of the 37 *S. aureus* isolates tested using the AST-GP79 card, 36 isolates (97.3%) exhibited resistance to at least two antimicrobials. The observed resistance rates, in descending order, were as follows: clindamycin (CLY) (100%), erythromycin (ERY) (80.6%), benzylpenicillin (PCG) (69.4%), tilmicosin (TIL) (52.8%), tylosin (TYL) (52.8%), tetracycline (TET) (52.8%), oxacillin (OXA) (50%), amikacin (AMK) (47.2%), ceftiofur (CTF) (16.7%), gentamicin (GEN) (8.3%), kanamycin (KAN) (8.3%), and cefalothin (CET) (2.8%). No resistance was recorded against ampicillin (AMP), cefquinome (CEF), neomycin (NEO), enrofloxacin (ENR), florfenicol (FLO), or trimethoprim-sulfamethoxazole (SXT). Among the tested *S. aureus* isolates, three strains showed the highest level of resistance, being resistant to 8 of the 18 antimicrobials tested from five classes, including CLY, ERY, PCG, TIL, TYL, TET, GEN, and KAN (Table 2).

The MDR profiles of the analyzed *S. aureus* strains are summarized in Table 2. A total of 33 multidrug-resistant *S. aureus* isolates, recovered from raw sheep milk, demonstrated diverse resistance profiles involving combinations of three to five antimicrobial classes.

The majority of isolates (15/33; 45.5%) exhibited resistance to five antimicrobial classes, most commonly involving lincomycins, macrolides, β-lactams, tetracyclines, and aminoglycosides. The most frequent resistance phenotype was CLY + ERY + PCG + TET + OXA + AMK, observed in 10 isolates. An additional 5 isolates (15.2%) showed resistance to four classes, and 13 isolates (39.4%) were resistant to three classes. Notably, lincomycin (CLY) and macrolides (ERY, TIL, TYL) were present in nearly all MDR patterns. Two additional *S. aureus* strains exhibited resistance to CLY, ERY, TIL, and TYL, while another strain was resistant to CLY and AMK. However, these isolates were not classified as MDR, as their resistance was limited to fewer than three antimicrobial classes, which does not meet the standard definition of MDR.

These results highlight a high prevalence of MDR among *S. aureus* isolates from sheep milk, emphasizing the potential risk for antimicrobial treatment failure and the need for enhanced surveillance in the dairy production chain.

## 3. Discussion

This study provides new insights into the occurrence and antimicrobial resistance profiles of *S. aureus* in raw sheep milk from traditional sheep farms in the Banat region of Romania. Of the 106 raw milk samples analyzed, CPS were detected in 65.1% and *S. aureus* was confirmed in 34.9% of all samples. The detection of CPS in more than half of the analyzed samples is a clear indicator of microbial contamination and potential udder health issues. CPS are frequently associated with subclinical mastitis in dairy animals and can enter milk through poor hygiene practices during manual milking or inadequate equipment sanitation [10,17]. Beyond being a marker of poor hygiene, CPS, including *S. aureus*, pose a food safety risk due to their ability to produce heat-stable enterotoxins, which can cause foodborne intoxications even after pasteurization [6,11]. The high prevalence observed in this study is consistent with earlier reports from Romanian artisanal dairy products [14] and international findings in traditional sheep milk production systems [5,18], underscoring the need for targeted hygiene interventions and pathogen monitoring in the raw milk value chain. Furthermore, although the enumeration of CPS in raw sheep milk is not currently defined as a hygiene or safety criterion under European Union regulations, the recorded contamination levels may significantly affect the microbiological quality and safety of derived dairy products, particularly those made from unpasteurized milk. According to Regulation (EC) No. 2073/2005, as amended by Regulation (EC) No. 1441/2007, when CPS levels exceed 10^5^ CFU/g in cheese made from raw milk, the product must be tested for the presence of staphylococcal enterotoxins due to the associated risk of foodborne intoxication [19].

The detection of *S. aureus* in over one-third of raw sheep milk samples is concerning from a food safety perspective, particularly given its potential to produce heat-stable enterotoxins. In Romania, especially in rural and traditional communities such as those in the Banat region, the consumption of unpasteurized sheep milk and artisanal dairy products remains common. These products are often consumed directly from the farm or sold in informal markets without undergoing thermal treatment. This practice increases the likelihood of exposure to viable pathogens and antimicrobial-resistant strains, thereby heightening the public health relevance of the findings reported in this study. This finding aligns with previous reports on traditional cheese and dairy products in Romania [14], reinforcing the need for stricter hygiene and monitoring practices. The recorded 34.9% prevalence rate is notably higher than the global pooled estimate for Caprinae milk (~25.8%; 95% CI: 17.5–35.0%) [20]. The rate aligns with data from Switzerland (33%) [21] and the Czech Republic (34.8%) [22], and is similar to the range reported in Greek sheep farms (24–63%) [23]. Studies specifically targeting animals with subclinical mastitis have revealed higher detection rates (e.g., 88.4% in Țurcana sheep in Romania [24], and 11.4% in Jordanian ewes [25]), but these differ in scope from bulk or retail milk studies. These comparisons suggest that our observed prevalence is consistent with upper-range contamination levels in conventional small ruminant milk systems. However, caution should be exercised when comparing the prevalence values recorded in this study with those reported in similar investigations, as differences in sampling strategy, herd size, geographical region, season, and laboratory methods can significantly influence the detection rates of *S. aureus* [17].

Species-level identification of *S. aureus* using the Vitek 2 system matched the molecular results (16S rDNA and *nuc* genes). This supports the reliability of combining phenotypic and genotypic approaches. While automated biochemical systems offer rapid and high-throughput identification, molecular confirmation using conserved and species-specific genetic markers provides an additional layer of diagnostic certainty, particularly in food safety and epidemiological investigations [9].

The detection of MRSA strains in raw sheep milk (13.5%) in the present study is of significant medical and public health concern. Livestock-associated MRSA (LA-MRSA) strains, such as those harboring the *mecA* gene, have been increasingly reported in both animal and human populations, particularly among individuals with close contact to livestock [12,13]. These strains can act as reservoirs for zoonotic transmission, either through direct exposure or via the food chain, particularly in raw milk or unpasteurized dairy products [11,26]. Moreover, the MDR commonly associated with MRSA limits available therapeutic options and increases the risk of treatment failure in both veterinary and human medicine [27]. Their persistence in the farm environment, often due to biofilm formation and poor hygiene practices, further exacerbates their epidemiological impact [10]. Similar findings have been reported in bovine mastitis in milk and dairy herds across Europe, where livestock-associated MRSA (LA-MRSA) poses a challenge to antimicrobial stewardship and infection control [13]. These findings underscore the importance of routine surveillance and responsible antimicrobial use in the dairy sector, particularly in traditional farming systems where biosecurity may be limited [28].

A striking 89.2% of *S. aureus* isolates demonstrated MDR, with resistance most commonly observed against clindamycin, erythromycin, penicillin, and tetracycline. When comparing the resistance profiles of *S. aureus* strains with those reported in a previous study by Morar et al. [14], which investigated isolates from traditional sheep cheeses marketed in the same region, the most striking difference is observed in AMK resistance: 90.1% in cheese-derived strains compared to only 47.2% in our raw milk isolates. Additionally, CLY and ERY resistance were substantially greater in our isolates (100% and 80.6%, respectively) compared to cheese-derived strains (30.6% and 22.4%). Conversely, PCG resistance is also elevated in our raw milk isolates (69.4%) relative to cheese isolates (53.1%), and OXA resistance, an indicator of MRSA, was three times higher in raw milk (50%) than in cheese-based isolates (16.3%). These discrepancies may reflect differences in antimicrobial use during lactation versus during cheese production, differential survival or selection pressure during processing, and microbial dynamics inherent to raw milk versus matured cheese matrices. Compared to international data, the *S. aureus* strains in our study exhibit substantially elevated resistance to several key antimicrobials. In Greece, CLY and PCG resistance among bulk-tank sheep milk isolates was considerably lower (17.7% and ~34%, respectively), and methicillin resistance was rare (~11.6%) [29]. In Southern Spain, resistance to TET (28.9%) and PCG (22.2%) was also much lower than in our isolates (52.8% and 69.4%), and no methicillin resistance was identified [30]. A Sicilian study of mastitic sheep milk reported moderate MDR (31%) but low *mecA* detection (<2%), in contrast to our 50% OXA resistance [31]. In Egypt’s Sohag Governorate, extremely high resistance rates (>90%) to AMK, PCG, and TET were noted in sheep milk, exceeding those in our findings, though our AMK resistance was lower (47.2% vs. 91.6%) [32]. These disparities likely reflect differences in antimicrobial usage policies, animal management practices, and regional veterinary treatment protocols.

Resistance to multiple antimicrobials was widespread among the isolates, with CLY (100%) and ERY (80.6%) resistance particularly dominant. These results are consistent with trends observed in other Romanian dairy sectors, including those reported by Pascu et al. [15], suggesting potential cross-resistance within β-lactams and macrolides due to historical overuse in veterinary medicine. Furthermore, the identification of 33 MDR strains, resistant to three or more antimicrobial classes, underscores the growing challenge of treating *S. aureus*-related infections and the risk of resistance transfer through the food chain. The most frequent MDR phenotype, CLY + ERY + PCG + TET + OXA + AMK, reflects resistance spanning five distinct antimicrobial classes and demonstrates the adaptive potential of these strains under antimicrobial pressure [10,27].

The high prevalence of CPS and MDR *S. aureus* in this study is likely influenced by traditional milking practices and hygiene conditions. Manual milking without prior teat disinfection, coupled with inadequate equipment sanitation, creates opportunities for both environmental contamination and sheep-to-sheep pathogen transmission [17,26]. These conditions are common in smallholder and traditional systems throughout Romania and other parts of Eastern Europe [7,24]. Accordingly, field observations in the present study revealed that manual milking was widely practiced without pre-milking teat disinfection or adequate cleaning of milking vessels. These practices are likely contributors to the elevated levels of CPS and *S. aureus* detected in this study. As a preventive measure, implementing basic hygiene protocols such as teat disinfection before milking, routine sanitation of milking equipment, and regular health checks of lactating ewes should be prioritized. These simple interventions could significantly reduce microbial contamination and the dissemination of antimicrobial-resistant pathogens in smallholder dairy systems. Therefore, improving milking hygiene, introducing farmer education programs, and reinforcing biosecurity protocols could substantially reduce the microbial load and limit the dissemination of resistant strains [10,15,33].

This study provides important insights into the prevalence and antimicrobial resistance of *Staphylococcus aureus* in raw sheep milk; however, it has several limitations. First, molecular characterization was restricted to the detection of *mecA*, *nuc*, and 16S rDNA genes. The absence of screening for *S. aureus*-specific virulence and enterotoxin genes (e.g., *sea*, *seb*, *sec*, *tst*) limits our ability to assess the toxigenic potential of the isolates and, consequently, the full extent of the public health risk. Incorporating these markers in future studies would offer a more comprehensive understanding of the pathogenic capacity of *S. aureus* strains in dairy environments. Second, the lack of data on antimicrobial usage practices on the sampled farms restricts our capacity to interpret resistance patterns in a broader epidemiological context. To strengthen causal inferences and elucidate transmission dynamics, future research should include farm-level treatment data, along with longitudinal surveillance and expanded molecular typing approaches.

## 4. Materials and Methods

### 4.1. Study Locations and Sampling Procedure

The investigation was conducted during the peak lactation periods of dairy ewes in April and May of 2024 and 2025 in Timiș County, part of the Banat region in western Romania. This region hosts one of the largest sheep populations at the county level, with approximately 700,000 sheep, representing around 7% of the national flock, raised on more than 2400 officially registered sheep farms (Sanitary Veterinary Directorate of Timiș County; reference date: April 2025).

To ensure statistically meaningful results, the minimum sample size was calculated using Cochran’s formula [34], assuming an expected prevalence of 84% based on recent data from a study on sheep-milk-origin dairy products [14], with an absolute precision of 7% and a confidence level of 95%. This yielded a required sample size of 106 milk samples.

A total of 106 dairy ewe farms, randomly selected and with the owners’ informed consent, participated in the study. These farms predominantly reared autochthonous breeds such as Țurcană, Țigaie, and Merino under extensive, pasture-based systems. Flock sizes ranged from 60 to 3000 animals. Sampling was conducted once per farm, immediately after early morning milking, with a total of 106 unique farms enrolled in the study. No farms was revisited in subsequent months or years to avoid duplication of data. Manual milking, without prior disinfection or cleaning of the teats, was performed in traditional sheepfolds, reflecting widespread national practices. Although considered outdated, manual milking remains common across Romanian farms due to limited adoption of automated systems [26].

From each farm, approximately 500 mL of raw milk was collected aseptically at the end of the milking process, directly from the milk storage vessels used on-site. Samples were transferred into sterile glass containers and transported under refrigerated conditions (≤4 °C) to the Microbiological Risk Assessment Laboratory, Faculty of Veterinary Medicine, Timișoara. Microbiological analyses for the detection of *S. aureus* were initiated within two hours after sample arrival, in accordance with standard diagnostic protocols.

### 4.2. Isolation, Quantification of CPS, and Identification of S. aureus

The detection and quantification of CPS were carried out in accordance with the ISO 6888-1:2021 guidelines, incorporating minor methodological adjustments [35]. To begin, each raw milk sample was thoroughly homogenized directly in its original sterile collection bottle. Serial decimal dilutions were then prepared in sterile 0.5% peptone water, reaching up to a 10^−4^ (1:10,000) dilution.

From each dilution level, 0.1 mL aliquots were plated onto Baird–Parker agar (Oxoid, Basingstoke, Hampshire, UK) supplemented with egg yolk tellurite emulsion, a selective medium tailored for the presumptive recovery of coagulase-positive staphylococci (CPS). Colonies with typical morphology were further confirmed by coagulase testing. The inoculated plates were incubated at 37 °C for 36 to 48 h. Colonies exhibiting the classical CPS morphology, meaning black, convex, circular growths with clear or opaque halos, were enumerated using manual colony-forming unit (CFU) counting techniques, enabling estimation of viable bacterial loads.

From each CPS-positive plate, five representative colonies were randomly chosen and subcultured on Brain Heart Infusion (BHI) agar (Biokar Diagnostics, Allone, France) and incubated at 37 °C for 24 h. The resulting isolates underwent preliminary identification through Gram staining and standard catalase and coagulase tests to confirm presumptive *Staphylococcus* spp. For species-level identification, isolates were analyzed using the Vitek 2 Compact automated system (bioMérieux, Marcy l’Etoile, France), employing the ID-GP identification cards, and processed according to the manufacturer’s instructions.

### 4.3. Molecular Identification and Characterization of S. aureus Isolates

A single *S. aureus* colony from each culture-positive specimen was selected for direct molecular analysis. Genomic DNA extraction was performed using the PureLink™ Genomic DNA Mini Kit (Invitrogen™, Carlsbad, CA, USA) in accordance with the manufacturer’s guidelines. The concentration and purity of the extracted genomic DNA were measured using a NanoDrop 2000 spectrophotometer (Thermo Fisher Scientific, Waltham, MA, USA), and purity was assessed by A260/A280 ratio. DNA integrity was confirmed by electrophoresis on a 1% agarose gel stained with MidoriGreen (Nippon Genetics^®^, Europe, GmbH, Düren, Germany). Only DNA samples with appropriate purity and intact bands were used for downstream PCR analyses. Initial molecular identification employed a genus-specific conventional uniplex PCR assay targeting the V4 hypervariable region of the 16S rDNA gene (~886 bp) using the primer pair 16S-1 (5′-GTGCCAGCAGCCGCGGTAA-3′) and 16S-2 (5′-AGACCCGGGAACGTATTCAC-3′) as described by Poulsen et al. [36].

Subsequently, to confirm species specificity, amplification of the *nuc* gene (~255 bp), encoding the thermostable nuclease characteristic of *S. aureus*, was conducted using the primer pair nuc-1 (5′-TCAGCAAATGCATCACAAACAG-3′) and nuc-2 (5′-CGTAAATGCACTTGCTTCAGG-3′). Resistance to methicillin was assessed by detecting the *mecA* gene (533 bp), responsible for synthesizing the penicillin-binding protein 2a (PBP2a), using mecA-1 (5′-GGGATCATAGCGTCATTATTC-3′) and mecA-2 (5′-AACGATTGTGACACGATAGCC-3′) primers. The primers used for amplification of the previously mentioned genes were adopted from Poulsen et al. [36], a previously validated study. The primer sequences and target gene regions were not modified or newly designed in this study.

All PCR mixtures and thermal cycling parameters followed the protocol described by Poulsen et al. [36]. Each assay included *S. aureus* ATCC25923™ harboring *mecA* as the positive control and PCR-grade water as a negative (no-template) control. Amplicons were visualized through electrophoresis on a 1.8% agarose gel stained with MidoriGreen (Nippon Genetics^®^, Europe, GmbH, Düren, Germany).

### 4.4. Antimicrobial Resistance Profiling of S. aureus Isolates Recovered from Raw Sheep Milk Using the Vitek2 System

A total of 37 *S. aureus* isolates derived from raw sheep milk were subjected to antimicrobial resistance testing using the fully automated Vitek2 system (bioMérieux, Marcy l’Etoile, France), utilizing the AST-GP79 diagnostic card. This specific card evaluates susceptibility against 18 antimicrobial compounds, encompassing a wide array of antimicrobial classes such as β-lactams, aminoglycosides, fluoroquinolones, macrolides, lincosamides, phenicols, sulfonamides, and tetracyclines. The Vitek2 platform automatically categorized each isolate as susceptible, intermediate, or resistant based on predefined minimum inhibitory concentration (MIC) thresholds.

The antimicrobials assessed included: PCG (MIC range 0.03–0.5 µg/mL), AMP (MIC range 0.25–16 µg/mL), OXA (MIC range 0.25–4 µg/mL), CET (MIC range 2–32 µg/mL), CTF (MIC range 0.5–8 µg/mL), CEF (MIC range 1–64 µg/mL), AMK (MIC range 2–64 µg/mL), GEN (MIC range 0.5–16 µg/mL), KAN (MIC range 4–64 µg/mL), NEO (2–32 µg/mL), ENR (MIC range 0.5–8 µg/mL), ERY (MIC range 0.25–8 µg/mL), TIL (MIC range 0.25–4 µg/mL), TYL (MIC range 1–32 µg/mL), CLY (MIC range 0.125–4 µg/mL), FLO (MIC range 4–32 µg/mL), TET (MIC range 1–16 µg/mL), and SXT (MIC range 10–320 µg/mL).

MDR was defined as acquired resistance to at least one antimicrobial in three or more antibiotic classes, following the criteria established by Magiorakos et al. [37]. Clinical breakpoints were interpreted according to the guidelines of the Clinical Laboratory Standards Institute (CLSI) [38]. Internal quality control for the antimicrobial testing procedures was ensured through the use of *S. aureus* ATCC^®^ 29213™ as the reference strain.

### 4.5. Statistical Analysis

All statistical evaluations were performed using SPSS version 23 (IBM Corp., Armonk, NY, USA). Variations in the differences in the frequency of isolation of *S. aureus* strains across different sampling months were assessed using one-way analysis of variance (ANOVA). Statistical significance was established at a threshold of *p* < 0.05.

## 5. Conclusions

This study provides compelling evidence of the widespread occurrence of *S. aureus*, MRSA and MDR strains, in raw sheep milk sourced from traditional farms in the Banat region of Romania. The detection of *S. aureus* in over one-third of samples, coupled with a high prevalence of AMR resistance, highlights a critical intersection between animal health, food safety, and public health. These findings raise particular concern given the continued production and consumption of unpasteurized dairy products in traditional settings, which may serve as a vehicle for the dissemination of AMR pathogens into the broader population.

To mitigate these risks, there is an urgent need for integrated surveillance programs that monitor AMR in the dairy sector, as well as the implementation of targeted interventions aimed at improving on-farm hygiene, milking practices, and the judicious use of antimicrobials in livestock. Strengthening these preventive measures is essential not only for protecting consumer health but also for preserving the efficacy of critical antimicrobial agents and promoting sustainable dairy farming practices.

Future research should include the routine screening of additional genetic markers, such as those encoding staphylococcal enterotoxins, virulence factors, and mobile genetic elements, to better assess the pathogenicity and transmission potential of *S. aureus* in raw milk. Such data would support more comprehensive risk assessments and help develop effective control measures to ensure the safety of traditionally produced dairy products.

## Figures and Tables

**Table 1:** Presence of CPS and *S. aureus* and their methicillin-resistant genotypic profiles in the investigated raw sheep milk samples from the Banat region, Romania.

Year	Month (Number Examined)	Number of Samples Containing CPS (%)	CPS Levels of Contamination (log CFU/g^−1^)			Number of CPS-Positive Samples Containing *S. aureus* (%)	Number of *S. aureus* with *mecA* Gene (%)
			min	max	Average (±Standard Deviation)		
2024	April (*n* = 19)	12 (63.2)	1.15	4.45	2.14 (±0.93)	7 (58.3)	1 (14.3)
	May (*n* = 27)	19 (70.4)	1.00	3.62	2.07 (±0.76)	8 (42.1)	-
2025	April (*n* = 23)	14 (60.9)	1.03	4.48	2.49 (±0.91)	8 (57.1)	2 (25.0)
	May (*n* = 37)	24 (64.9)	1.05	4.91	2.10 (±0.94)	14 (58.3)	2 (14.3)
Overall	*n* = 106	69 (65.1)	N.A.	N.A.	2.20 (±0.20)	37 (53.6)	5 (13.5)

Legend: CPS—coagulase-positive staphylococci.

**Table 2:** The combination of multidrug-resistant phenotypes observed in the tested *S. aureus* isolates.

Number of Isolates	Classes with Resistance	Antimicrobial Resistance Profile of *S. aureus* Strains (Number)
10	lincomycins, macrolides, ß-lactams, tetracyclines, aminoglycosides	CLY + ERY + PCG + TET + OXA + AMK (6)
3	lincomycins, macrolides, ß-lactams, tetracyclines, aminoglycosides	CLY + ERY+ PCG + TIL + TYL + TET + GEN + KAN (8)
1	lincomycins, macrolides, ß-lactams, tetracyclines, aminoglycosides	CLY + ERY + TET + OXA + AMK (5)
1	lincomycins, macrolides, ß-lactams, tetracyclines, aminoglycosides	CLY + ERY + PCG + TET + AMK (5)
2	lincomycins, macrolides, ß-lactams, tetracyclines	CLY + ERY+ PCG + TIL + TYL + TET (6)
1	lincomycins, macrolides, ß-lactams, tetracyclines	CLY + ERY+ PCG + TIL + TYL + TET + CET (7)
1	lincomycins, macrolides, ß-lactams, aminoglycosides	CLY + ERY + PCG + OXA + AMK (5)
1	Lincomycins, macrolides, ß-lactams, aminoglycosides	CLY + ERY + OXA + AMK (4)
4	lincomycins, macrolides, ß-lactams	CLY + ERY+ PCG + TIL + TYL + OXA + CTF (7)
2	lincomycins, macrolides, ß-lactams	CLY + PCG + TIL + TYL + OXA + CTF (6)
2	lincomycins, macrolides, ß-lactams	CLY + PCG + TIL + TYL (4)
2	lincomycins, macrolides, tetracyclines	CLY + TIL + TYL + TET (4)
3	lincomycins, macrolides, amynoglycosides	CLY + ERY + AMK (3)

Legend: PCG—benzylpenicillin, OXA—oxacillin, CET—cefalothin, CTF—ceftiofur, AMK—amikacin, GEN—gentamicin, KAN—kanamycin, ERY—erythromycin, TIL—tilmicosin, TYL—tylosin, CLY—clindamycin, TET—tetracycline.

## Data Availability

All data generated or analyzed during this study are included in the submitted version of the manuscript.
